# PHF6 regulates cell cycle progression by suppressing ribosomal RNA synthesis

**DOI:** 10.1186/1756-8935-6-S1-P134

**Published:** 2013-04-08

**Authors:** Jiadong Wang, Justin Wai Chung Leung, Zihua Gong, Lin Feng, Xiaobing Shi, Junjie Chen

**Affiliations:** 1 Department of Experimental Radiation Oncology, The University of Texas MD Anderson Cancer Center, 1515 Holcombe Boulevard, Houston, Texas 77030, USA; 2 Department of Biochemistry and Molecular Biology, The University of Texas MD Anderson Cancer Center, 1515 Holcombe Boulevard, Houston, Texas 77030, USA

## 

Mutation of PHF6, which results in X-linked mental retardation disorder-Börjeson-Forssman-Lehmann syndrome, is also present in about 38% of adult T-cell acute lymphoblastic leukemias and 3% of adult acute myeloid leukemias. However, it remains to be determined exactly how PHF6 acts *in vivo* and what functions of PHF6 may be associated with its putative tumor suppressor function. Here, we demonstrate that PHF6 is a nucleolus, ribosomal RNA promoter-associated protein. PHF6 directly interacts with UBF through its PHD1 domain and suppresses rRNA transcription by affecting the protein level of UBF. Knockdown of PHF6 impairs cell proliferation and arrests cells at G2/M phase, which is accompanied by increased level of phosphorylated H2AX (γ-H2AX), indicating that PHF6 deficiency leads to the accumulation of DNA damage in the cell. We found that increased DNA damage occurs at rDNA locus in PHF6-deficient cells. This effect could be reversed by knocking-down UBF or over-expressing RNASE1, which removes RNA:DNA hybrids, suggesting that there is a functional link between rRNA synthesis and genomic stability at rDNA locus. Together, these results reveal that the key function of PHF6 is involved in regulating rRNA synthesis, which may contribute to its roles in cell cycle control, genomic maintenance and tumor suppression.

